# Transplantation of Neural Precursor Cells Attenuates Chronic Immune Environment in Cervical Spinal Cord Injury

**DOI:** 10.3389/fneur.2018.00428

**Published:** 2018-06-08

**Authors:** Lennart Riemann, Alexander Younsi, Moritz Scherer, Guoli Zheng, Thomas Skutella, Andreas W. Unterberg, Klaus Zweckberger

**Affiliations:** ^1^Department of Neurosurgery, Heidelberg University Hospital, Heidelberg, Germany; ^2^Department of Neuroanatomy, Institute for Anatomy and Cell Biology, Heidelberg University, Heidelberg, Germany

**Keywords:** spinal cord injury, stem cells, neural precursor cells, neuroregeneration, chronic inflammation, macrophages, apoptosis

## Abstract

Inflammation after traumatic spinal cord injury (SCI) is non-resolving and thus still present in chronic injury stages. It plays a key role in the pathophysiology of SCI and has been associated with further neurodegeneration and development of neuropathic pain. Neural precursor cells (NPCs) have been shown to reduce the acute and sub-acute inflammatory response after SCI. In the present study, we examined effects of NPC transplantation on the immune environment in chronic stages of SCI. SCI was induced in rats by clip-compression of the cervical spinal cord at the level C6-C7. NPCs were transplanted 10 days post-injury. The functional outcome was assessed weekly for 8 weeks using the Basso, Beattie, and Bresnahan scale, the CatWalk system, and the grid walk test. Afterwards, the rats were sacrificed, and spinal cord sections were examined for M1/M2 macrophages, T lymphocytes, astrogliosis, and apoptosis using immunofluorescence staining. Rats treated with NPCs had compared to the control group significantly fewer pro-inflammatory M1 macrophages and reduced immunodensity for inducible nitric oxide synthase (iNOS), their marker enzyme. Anti-inflammatory M2 macrophages were rarely present 8 weeks after the SCI. In this model, the sub-acute transplantation of NPCs did not support survival and proliferation of M2 macrophages. Post-traumatic apoptosis, however, was significantly reduced in the NPC group, which might be explained by the altered microenvironment following NPC transplantation. Corresponding to these findings, reactive astrogliosis was significantly reduced in NPC-transplanted animals. Furthermore, we could observe a trend toward smaller cavity sizes and functional improvement following NPC transplantation. Our data suggest that transplantation of NPCs following SCI might attenuate inflammation even in chronic injury stages. This might prevent further neurodegeneration and could also set a stage for improved neuroregeneration after SCI.

## Introduction

Cervical spinal cord injury (SCI) is a devastating event with severe consequences for patients including paralysis, autonomic dysfunctions, and sensory deficits. Less than 1% of spinal cord injury patients regain a normal neurological status by the time they are discharged from the hospital, and therapeutic options are still very limited (1, 2). The cervical spine is involved in 54% of spinal cord injuries—by far more often than any other region of the spine (1). From a translational point of view, animal models of cervical trauma are therefore highly clinically relevant.

Traumatic SCI induces an inflammatory response that includes immune cell infiltration, up-regulation of inflammatory cytokines and production of oxygen/nitrogen radicals (3, 4). Although some beneficial effects have been described, the resulting inflammatory milieu is regarded to impede neuroregeneration and to exacerbate secondary spinal cord damage (5). Moreover, post-traumatic inflammation is non-resolving and persists also in chronic stages of SCI (6–8).

While neutrophils disappear after 5–10 days following SCI, the numbers of both T lymphocytes and microglia/macrophages are chronically increased in the injured spinal cord (6, 7). T lymphocytes are part of a complex adaptive immune response with both beneficial and detrimental effects, though their occurence appears to be highly dependent upon species and staining method (9–11). Microglia/macrophages are the predominant inflammatory cell population after central nervous system (CNS) injuries (12). Their activation is associated with tissue damage, whereas the depletion of macrophages reportedly leads to improved recovery (13, 14).

SCI activates microglia/macrophages along a continuum of different phenotypes, which represent functional states (15). Two phenotypes of microglia/macrophages seem to play a key role for insufficient neuroregeneration after SCI: M1 macrophages, also termed classically activated, are part of the acute inflammatory response, but remain chronically activated (12, 16). They facilitate inflammation by secreting reactive oxygen species and pro-inflammatory cytokines and are suggested to be neurotoxic and detrimental to recovery after SCI (12, 16, 17). M2 macrophages, also termed alternatively activated, are thought to be anti-inflammatory, as they secrete immunosuppressive cytokines and possibly promote axonal growth (12, 16, 17). M2 macrophages are reported to greatly decrease in number over time after SCI (12).

Transplantation of stem cells is a promising strategy to improve neuroregeneration and functional recovery after SCI. Stem cells are likely to be effective through a variety of mechanisms including the secretion of growth factors and modulation of the hostile post-traumatic microenvironment (2, 18, 19). Importantly, stem cells may impact neuroinflammatory processes: It has been shown that transplantation of mesenchymal stem cells leads to a shift from the M1 to the M2 phenotype during the acute phase following SCI (20).

Neural precursor cells (NPCs) are promising candidates for stem cell transplantation, as they can differentiate into neurons, astrocytes, and oligodendrocytes, thus supporting regeneration and recovery (21–23). It has been reported that NPCs enhance remyelination, release neurotrophic factors, and support functional recovery (24–26). Additionally, NPCs seem to be able to modify the immune environment as they inhibit the activation of M1 macrophages and reduce the secretion of pro-inflammatory cytokines in the sub-acute phase after SCI (27).

In this study, we examined the effects of NPC transplantation on the immune environment at chronic stages of a clinically highly relevant cervical (C6-C7) SCI model.

## Materials and methods

### Animals and cervical spinal cord injury

A total of 22 female Wistar rats (250 g; Charles River Laboratories, Sulzfeld, Germany) were divided into a NPC (*n* = 13) and a control group (*n* = 9). All experimental protocols were approved by the Animal Care Committee of Heidelberg University. The contusion/compression model was performed with an aneurysm clip as previously described (28–30). Briefly, rats were anesthetized with isoflurane (2–2.5%) and a 1:1 mixture of O_2_ and N_2_O before a microsurgical laminectomy was performed at the C6/C7 level. A modified 28-g aneurysm clip (Fehlings Laboratory, Toronto, Canada) was applied extradurally, using a quick-release applicator for 1 min at the C6 level. The animals were subject to extensive post-operative care and received buprenorphine (0.05 mg/kg subcutaneously) and meloxicam (2.0 mg/kg) for 3–5 days. Fluids and nutritional support were administered to all injured animals. An antibiotic prophylaxis (moxifloxacin, 4 mg/kg) was given for 7 days, and bladders were manually expressed three times per day until the return of the bladder function. Animals were housed in a 12-h light-dark cycle at 26°C with food and water *ad libitum*.

### Isolation and culture of neural precursor cells (NPCs)

NPCs were isolated from the subventricular zone of 2-week-old embryos of green fluorescent protein (GFP) expressing transgenic Wistar rats. Tissue pieces from the cortical hemispheres free from meninges were obtained and washed in 2 mL cold PBS. After removing the buffer, 1.5 mL 0.05% trypsin/ethylenediaminetetraacetic acid with 0.2% deoxyribonuclease I per 10 tissue pieces was added. The suspension was then incubated at 37°C for 5 min before the enzymatic activity was inhibited by adding 10% fetal bovine serum. Additionally, the tissue was mechanically dissociated into a cell suspension with a fire-polished pipette before centrifuging the suspension for 6 min. NPCs were plated in poly-l-ornithine-laminin-coated tissue culture plates at a density of 1.5 × 10^4^ cells/cm^2^ in 1.5 mL growth medium containing Dulbecco's Modified Eagle's Medium/F12 with sodium bicarbonate and L-glutamine, 1% penicillin/streptomycin, 1× N2 supplement, 20 ng/mL bFGF, and 10 ng/mL EGF. Cells were incubated in a humidified incubator at 37°C with 5% CO_2_. When reaching a confluence of 80–90%, NPCs were split 1:3 to 1:6. For transplantation, the NPCs were washed once with PBS before incubation with Accutase for 3 min. The growth medium as described above was added, and detached cells were collected for transplantation.

### NPC transplantation

Prior to transplantation, NPCs were successfully characterized by colocalization with the NPC-marker Nestin (GFP+/Nestin+/DAPI+). Then, 10 days after SCI, animals were randomized into two groups (NPC or control), anesthetized as described above, and the dura of the spinal cord was exposed again under microscope observation. NPCs were transplanted into the spinal cord in four sites, bilaterally 2 mm rostral and caudal to the epicenter of the lesion, using a stereotactic microinjector (31). 4 × 10^5^ NPCs in 8 μL (i.e., 2 μL per site) of growth medium were injected into the spinal cord with a Hamilton syringe at a rate of 5 nL/s, 1.5 mm deep from the dorsal surface of the spinal cord. The needle was left in the spinal cord for an additional minute to allow for diffusion of the cells. Rats of the control group received the same amount of growth medium but without NPCs.

For a continuous administration of growth factors, an osmotic micropump (Alzet, Cupertino, USA; model 1007D) was subcutaneously implanted and connected to a microcatheter that was placed subdurally with its open tip over the epicenter of the lesion. Animals of both groups received intrathecally growth factors (PDGF-AA, 1 μg/100 μL; EGF, 3 μg/100 μL; bFGF, 3 μg/100 mL; all Sigma-Aldrich) diluted in 0.1% rat serum albumin for 7 days (30, 32).

### Animal perfusion and tissue processing

Eight weeks after SCI, the animals were deeply anesthetized with isoflurane and transcardially perfused with 50 mL 0.1 M cold PBS followed by 150 mL 4% paraformaldehyde in 0.1 M PBS (pH 7.4). Tissues were post-fixed in 4% paraformaldehyde for 24 h and then cryoprotected in 30% sucrose for 48 h. Spinal cord segments of 2 cm length centered around the injury site were dissected and embedded in mounting media on dry ice. Serial cryostat cross-sections (240 μm apart) were cut with a thickness of 30 μm, and slides were stored at −80°C until further processing.

### Immunofluorescence staining

For all immunofluorescence stainings, the slides/cells were first blocked with a blocking solution containing 5% non-fat milk, 1% bovine serum albumine, and 0.3% Triton-X100 in 0.1 M PBS for 1 h. The following primary antibodies, diluted in the same blocking solution, were then applied overnight: anti-Nestin (1:400, Millipore) for NPCs, anti-Iba1 (1:1000, Wako) for microglia/macrophages, anti-iNOS (1:500, Abcam) for M1 macrophages, anti-CD206 (1:500, R&D Systems) for M2 macrophages, anti-CD3 (1:100, Serotec) for T lymphocytes, anti-GFAP (1:400, Millipore) for astrocytes, and anti-caspase-3 (1:200, Cell Signaling) for apoptotic cells. Isotype controls with non-specific immunoglobulin at the same concentration were performed to ensure specificity of the antibody stainings (images not shown). Alexa Fluor 568 goat anti-mouse (1:400, Invitrogen), Alexa Fluor 647 goat anti-rabbit (1:400, Invitrogen), and Alexa Fluor 405 donkey anti-goat (1:400, Abcam) diluted in blocking solution without Triton-X100 were used as secondary antibodies and applied for 1 h before covering the slides with mounting medium.

### Image analysis and quantification

All images were obtained using a confocal laser microscope (LSM 700, Carl-Zeiss). Cell counting, as well as all immunodensity measurements, were performed by three independent investigators blinded to treatment groups.

To assess NPC survival, the areas with GFP-positive cells were traced under 10x magnification on each tissue section (transplant area). Considering transplant area and distance between the slides (240 μm), the total transplant volume was approximated using Cavalieri's method. For each tissue section, surviving NPCs (GFP+/DAPI+), neuronally differentiated NPCs (GFP+/DAPI+/NeuN+), and oligodendroglially differentiated NPCs (GFP+/DAPI+/APC+) were counted under 40 × magnification in 10 fields (368 × 368 μm^2^), which were randomly placed in the transplant area, and the mean density (in cells/mm^3^) for each field was calculated. The total number of surviving and differentiated NPCs was estimated by multiplying the averaged mean density with the transplant volume in each rat.

For cell counting of M1/M2 macrophages and T cells, seven perilesional sections (− 3, − 2, − 1, 0, 1, 2, and 3 mm from the epicenter) were selected from each animal. Cells were manually counted in five fields (368 × 368 μm^2^), which were always placed in the same topographical regions of the spinal cord: Bilaterally in the anterior and lateral funiculi and centrally in the posterior funiculus. M1 macrophages were defined as Iba1+/iNOS+/CD206-, M2 macrophages as Iba1+/CD206+/iNOS-, and T lymphocytes as DAPI+/CD3+ cells.

Quantification of caspase-3-positive cells was also performed on seven perilesional sections. The following ImageJ2 (National Institute of Health, Bethesda, MD, USA) algorithm was used for both the entire spinal cord and the gray matter: First, a Gaussian-filter (Sigma: 2.00) was applied to reduce background noise. The IsoData-thresholding algorithm was then used to transform the selected area (spinal cord/gray matter) into a binary image, in which only caspase-3-stained nuclei with signals above the threshold were displayed. Caspase-3-positive cells were then automatically counted using the “Analyze Particles” function. Only structures with a minimum area of 20 μm^2^ were counted to avoid inclusion of artifacts.

Immunodensity measurements of iNOS were performed on seven perilesional sections, whereas GFAP staining was measured on 5 perilesional sections (at the epicenter, 1.2 mm and 2.4 mm caudal and rostral). As described previously (30, 33), the entire spinal cord, the gray matter, and the cavity were traced separately with ImageJ. The parameters “Integrated density” and “Area” of the cavity were measured and subtracted from the respective values of the entire spinal cord and gray matter. This “subtracted integrated density” was then divided by the respective “subtracted area” yielding the immunodensity of the entire spinal cord and the gray matter for every staining. Results were averaged by distance and group or pooled by group as overall group means.

To assess cyst dimensions, GFAP was visualized on serial tissue sections (240 μm apart) and photographed at 10x magnification. When present, the cystic cavitation was manually measured using ImageJ. The total cyst volume was then calculated using Cavalieri's method, considering the total cyst area sum and the distance between slides.

### Behavioral assessment

In order to assess the functional outcome, the Basso-Beattie-Bresnahan locomotor rating scale (BBB) was performed on a weekly basis from 1 week before until 8 weeks after SCI by two independent observers blinded to treatment groups. For this test, rats were placed into an open field for 4 min, and hindlimb locomotor function, joint movement, coordination, and weight support were evaluated using a rating scale from 0 to 21 points (34). A BBB score of 21 indicates normal motor function, while a score of 0 is equivalent to complete paralysis.

In order to assess regeneration of forelimbs, footprint analysis as well as kinematic analysis was performed weekly using the CatWalk XT gait analysis system which allows an objective analysis of gait parameters (35). In short, footprints of animals crossing the CatWalk XT walkway illuminated by a fluorescent light were detected and recorded by a high-speed camera. Three runs were recorded for each animal and automatically analyzed by the CatWalk XT software. Measured parameters of all runs were then averaged per animal.

Fine motor coordination was tested 8 weeks after SCI by the grid walk test. In short, step errors of animals crossing a pathway of irregularly placed metal grids were counted over four runs and errors were averaged (36).

### Statistical analysis

Results are given as mean ± standard error of the mean. Differences between the NPC and the control group were evaluated for statistical significance using unpaired two-sample *t*-tests. A *p*-value of *p* < 0.05 was considered significant. All statistical analyses were conducted using the software R (37).

## Results

### Long-term survival and differentiation of NPCs

**Figure 1:**
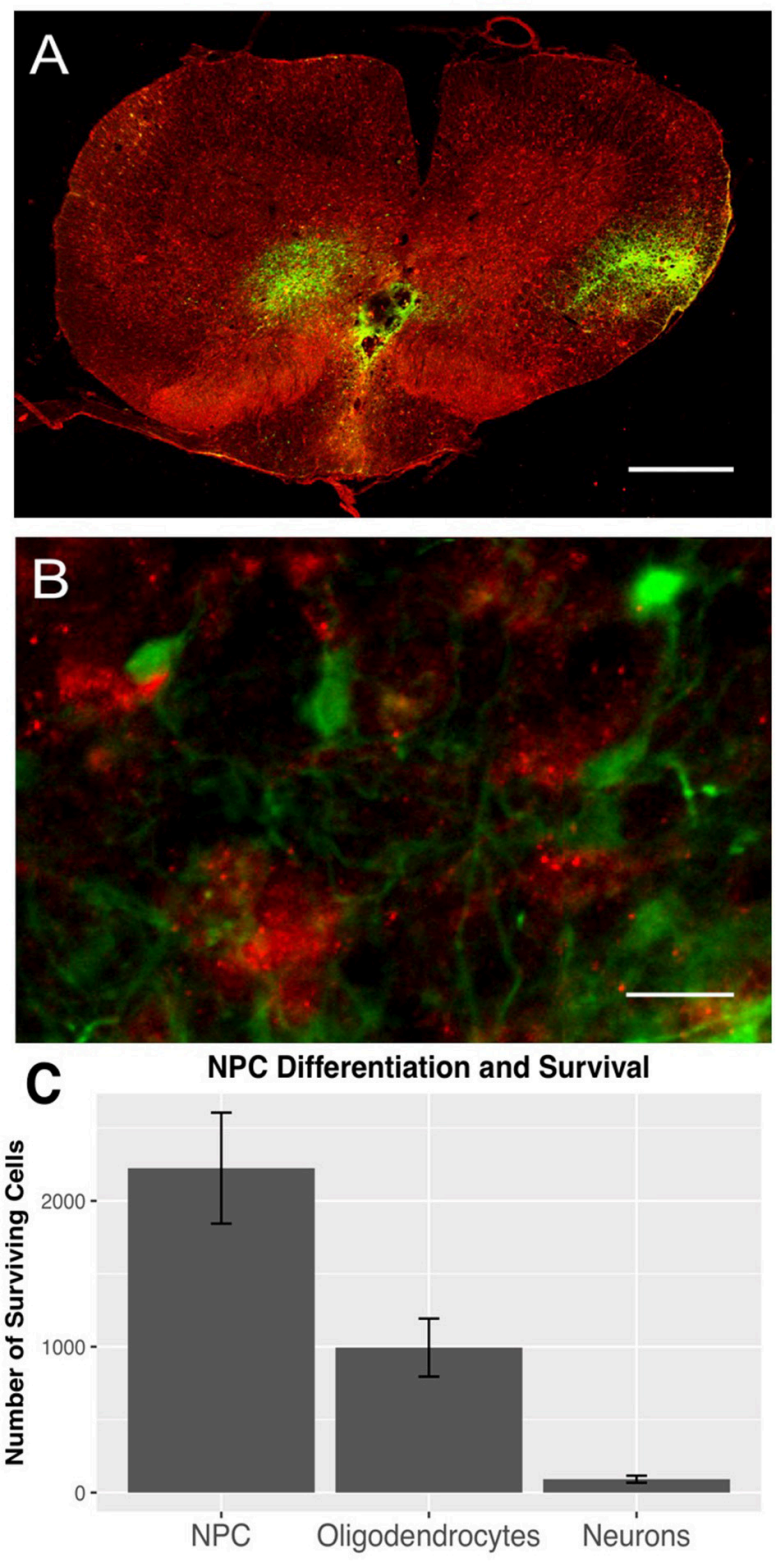
GFP-positive NPCs (green) and Iba1-positive macrophages (red) in the injured spinal cord 8 weeks after traumatic cervical SCI (*n* = 7). **(A)** GFP-positive NPCs were mainly distributed in the dorsal white and gray matter (10 × magnification, scale bar = 500 μm). **(B)** Additionally, GFP-expressing NPCs were often located very close to Iba1-positive macrophages (40 × magnification, scale bar = 15 μm). **(C)** Surviving NPCs differentiated primarily along the oligodendroglial lineage (GFP/APC), while only a minority of NPCs differentiated into neurons (GFP/NeuN).

To evaluate survival, differentiation, and distribution of transplanted NPCs, we quantified GFP-positive cells 6 weeks after transplantation (*n* = 11). The mean number of surviving NPCs definded as GFP+/DAPI+ was 2224.38 ± 380.37. All rats showed a substantial rostro-caudal distribution of NPCs over a length of 4.63 ± 0.41 mm, suggesting an outward migration of these cells from the transplant zone. The transplanted cells were usually located in the dorsal white or gray matter. Furthermore, we could observe a close spatial relationship between NPCs and macrophages (Figures 1A,B).

Surviving NPCs differentiated primarily along the oligodendroglial lineage (994.32 ± 199.03 GFP+/APC+), while only a minority of NPCs differentiated into neurons (91.91 ± 24.02 GFP+/NeuN+; Figure 1C).

### Assessment of macrophage polarization into an M1 and M2 phenotype

**Figure 2:**
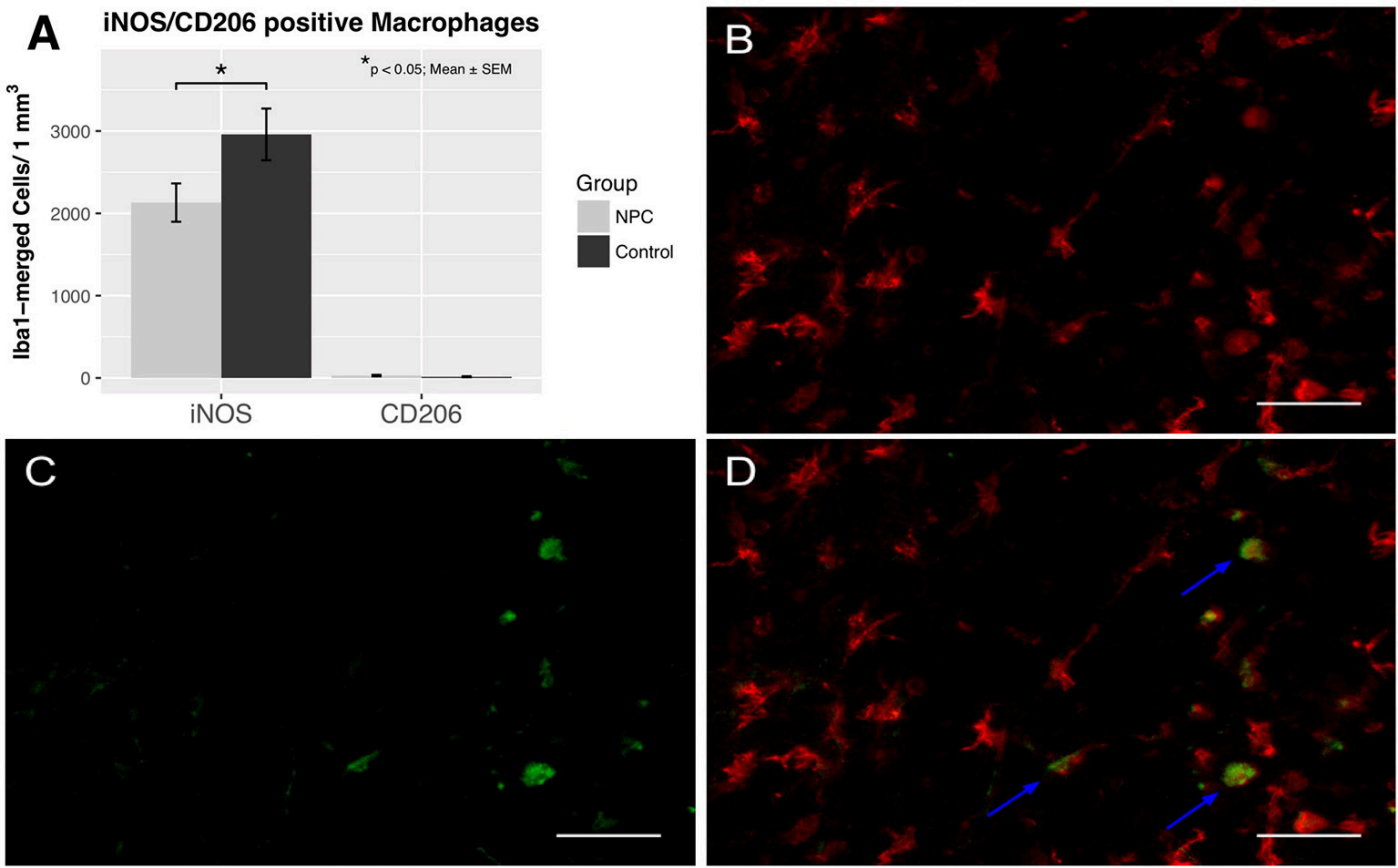
Quantification of M1 and M2 activation 8 weeks after SCI (*n* = 6–7). **(A)** Pro-inflammatory M1 macrophages were significantly reduced in the NPC group compared to the control group, while anti-inflammatory M2 macrophages were rarely observed in either group. **(B–D)** Colocalization of Iba1-positive macrophages (red) with the marker enzyme iNOS (green) indicated by the blue arrows confirm the presence of M1 activated macrophages (40 × magnification, scale bar = 40 μm).

To assess the effects of NPC transplantation on macrophage differentiation in chronic stages of the injury (i.e., 8 weeks after SCI), spinal cord sections were stained for Iba1, a marker for macrophages, iNOS, a marker for pro-inflammatory M1 macrophages, and CD206, a marker for anti-inflammatory M2 macrophages (control group, *n* = 6; NPC group, *n* = 7). Only M1 macrophages, but not M2 macrophages were observed in substantial numbers in both groups (Figure 2). M1 macrophage counts were significantly lower in the NPC group compared to the control group without stem cell transplantation (2,130 ± 233 vs. 2,959 ± 314 cells/mm^3^ for NPC and control group, respectively; *p* < 0.05). The number of M2 macrophages was very low without any significant group differences (29 ± 9 vs. 15 ± 6 cells/mm^3^ for NPC and control group, respectively).

### Assessment of iNOS activity in M1 macrophages

**Figure 3:**
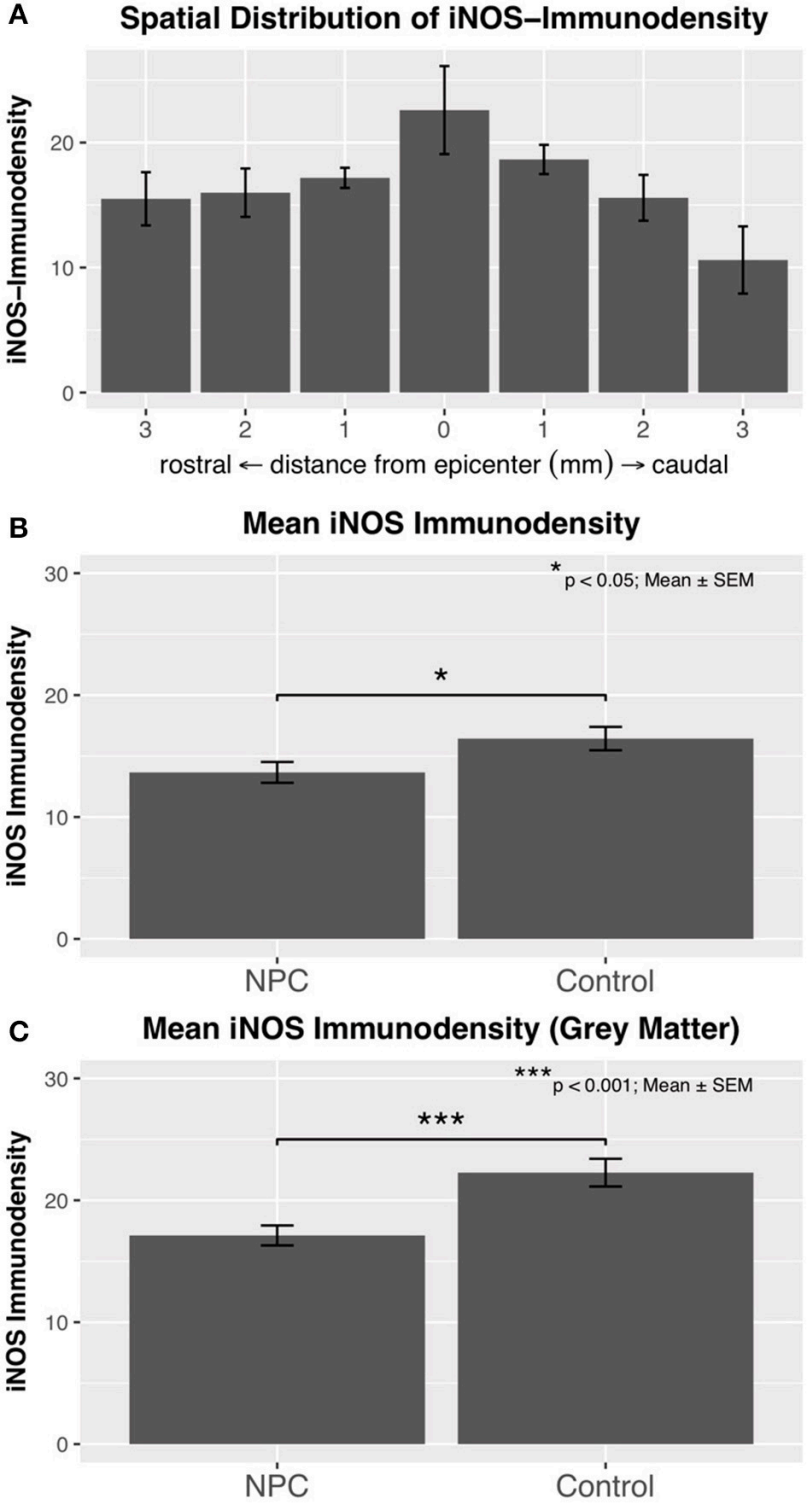
**(A)** Immunodensity of iNOS is in injured control animals (*n* = 6) maximal at the SCI epicenter with a gradual decline in intensity toward the periphery. **(B)** Mean iNOS immunodensity was significantly reduced in the NPC group compared to the control group, indicating a reduced pro-inflammatory M1 activation. **(C)** Immunodensity values for iNOS were especially high in the gray matter with a highly significant group difference in favor of the NPC group.

After confirmation that M1 macrophages are present in chronic stages of SCI, their functional activity and spatial distribution were evaluated by measuring the immunodensity of iNOS, a marker enzyme for M1 macrophages (control, *n* = 6; NPC, *n* = 7). This analysis revealed a chronic pro-inflammatory state in injured rats 8 weeks after SCI that was most distinct in the epicenter of the lesion (Figure 3A). The maximum of measured iNOS immunodensities was detected in the SCI epicenters of control animals with these values gradually decreasing toward the periphery. The mean immunodensity of perileserional sections was significantly lower in the NPC group compared to the control group (*p* < 0.05; Figure 3B). The immunodensity in the gray matter was generally higher compared to that in the entire spinal cord (Figure 3C). The difference in the mean iNOS immunodensity of the gray matter was highly significant between NPC and control group (*p* < 0.001).

### Presence of T lymphocytes

**Figure 4:**
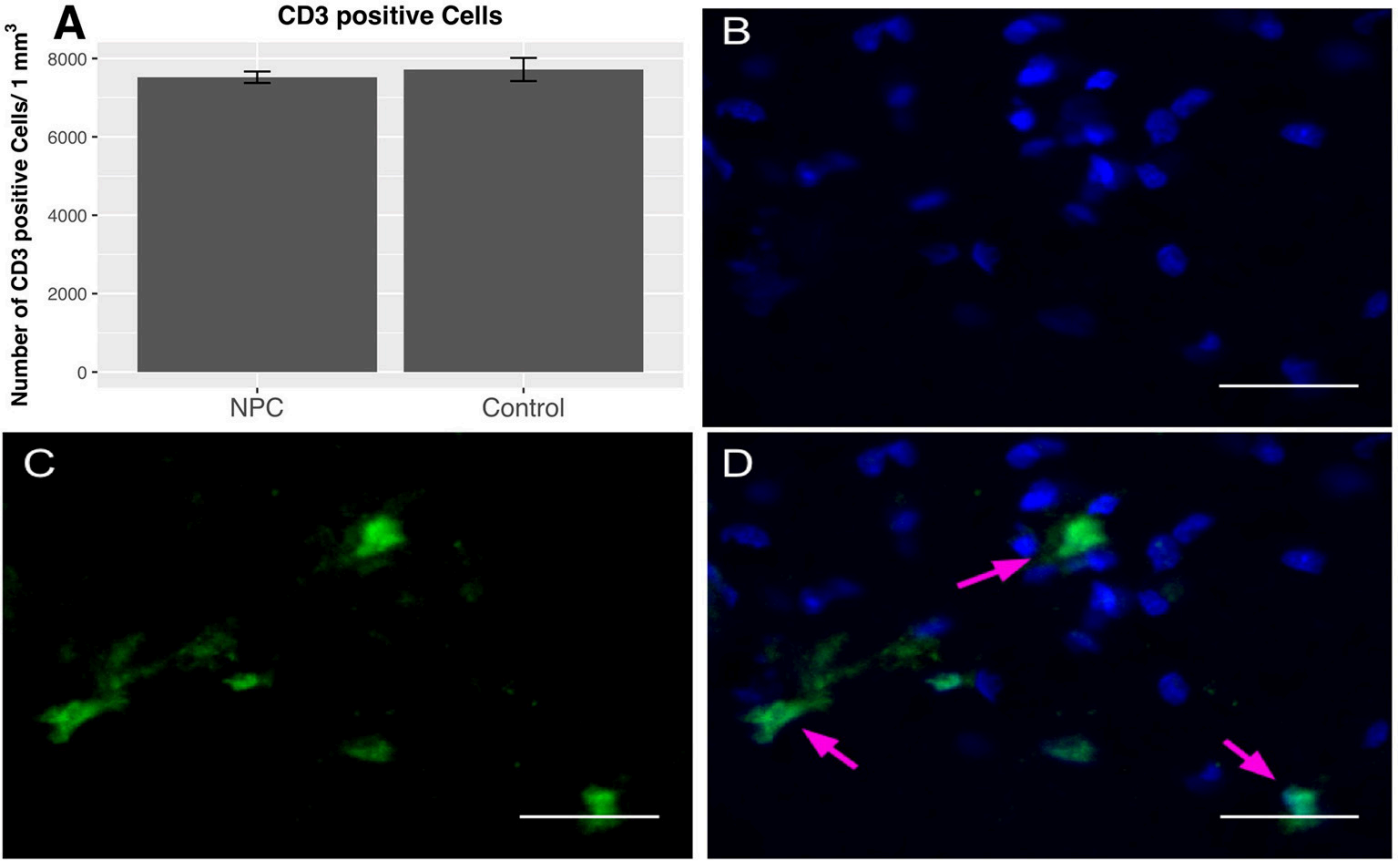
Quantification of CD-3-positive T lymphocytes 8 weeks after SCI (*n* = 5). **(A)** No group differences in perilesional total T cell numbers were observed between NPC and control group. **(B–D)** Confocal fluorescence microscopy (40 × magnification, scale bar = 15 μm) of the injured spinal cord showing CD-3-positive T lymphocytes (green) co-expressing the nuclear cell marker DAPI (blue).

To quantify the number of T lymphocytes, CD3-positive cells were counted 8 weeks post-injury (*n* = 5). As a part of the adaptive immune response, T lymphocytes are very rare in the uninjured spinal cord (0-3 cells/section) (10). Our data confirm the previously reported presence of T lymphocytes in substantial numbers during the chronic phase after SCI. We determined 7,520 ± 147 cells/mm^3^ in the NPC group and 7,717 ± 295 cells/mm^3^ in the control group; however, this finding does not show any significant differences between the treatment groups (Figure 4).

### Assessment of reactive astrogliosis

**Figure 5:**
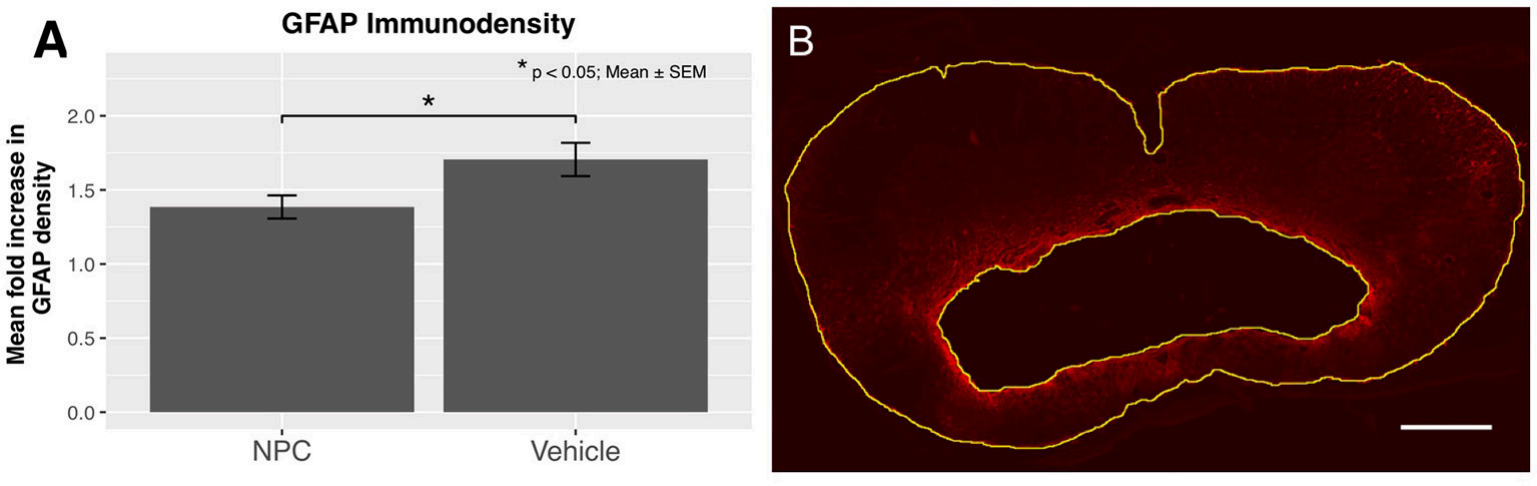
**(A)** The quantification of GFAP immunodensity 8 weeks after SCI shows a significant reduction of astrogliosis in the NPC group (*n* = 5). **(B)** Confocal fluorescence microscopy (10 × magnification, scale bar = 500 μm) showing GFAP-positive astrocytes (red) with a high density near the cavitation. The outskirts of the astrogliosis, as well as the edge of the cystic cavitation, are shown in yellow.

Reactive astrogliosis, a hallmark of CNS pathologies, is closely related to inflammation. Astrocytes are involved in the regulation of CNS inflammation, while also producing pro-inflammatory cytokines and reactive oxygen species (38). We examined the expression of GFAP to evaluate the extent of reactive astrogliosis 8 weeks after SCI (*n* = 5). In the NPC group, a significant decrease in the mean GFAP immunodensity compared to the control group was observed, indicating a significantly reduced astrogliosis (*p* < 0.05; Figure 5A).

### Analysis of the intramedullary cyst size

**Figure 6:**
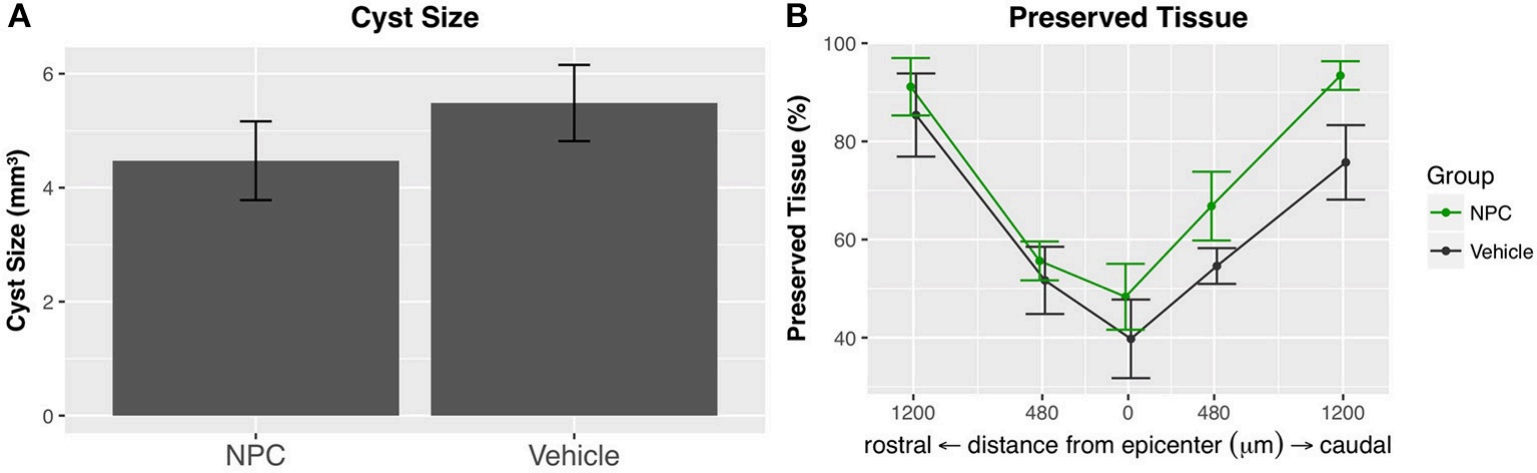
**(A)** The intramedullary cavity size, assessed on GFAP-stained cross-sections (see Figure 5B), was reduced in the NPC group compared to control, although this difference did not reach statistical significance (*n* = 5). **(B)** The preserved tissue as a percentage of the entire spinal cord volume was higher in the NPC group at all measured time points without reaching statistical significance.

The formation of an intramedullary cavity is a major obstacle for regenerating axons in both humans and rats (39). We used GFAP-stained sections to quantify the volume of the post-traumatic cystic cavitation (*n* = 5; Figure 5B). The mean cyst size was reduced by about 18% in the NPC group compared to the control group without reaching statistical significance (NPC, 4.47 ± 0.69 mm^3^; control, 5.48 ± 0.67 mm^3^; Figure 6A). At the epicenter, the proportion of preserved tissue was 51.67 ± 6.84% in the control group vs. 66.81 ± 7.01% in the NPC group. In sections 1.2 mm rostral and caudal from the epicenter, 91.13 ± 5.85% and 93.41 ± 2.91% of cord tissue was intact in the NPC group compared to 85.37 ± 8.47% and 75.71 ± 7.59% in the control group, respectively (Figure 6B). All measured parameters demonstrated a benefit of NPC transplantation; however, the differences did not reach statistical significance.

### Assessment of apoptosis

**Figure 7:**
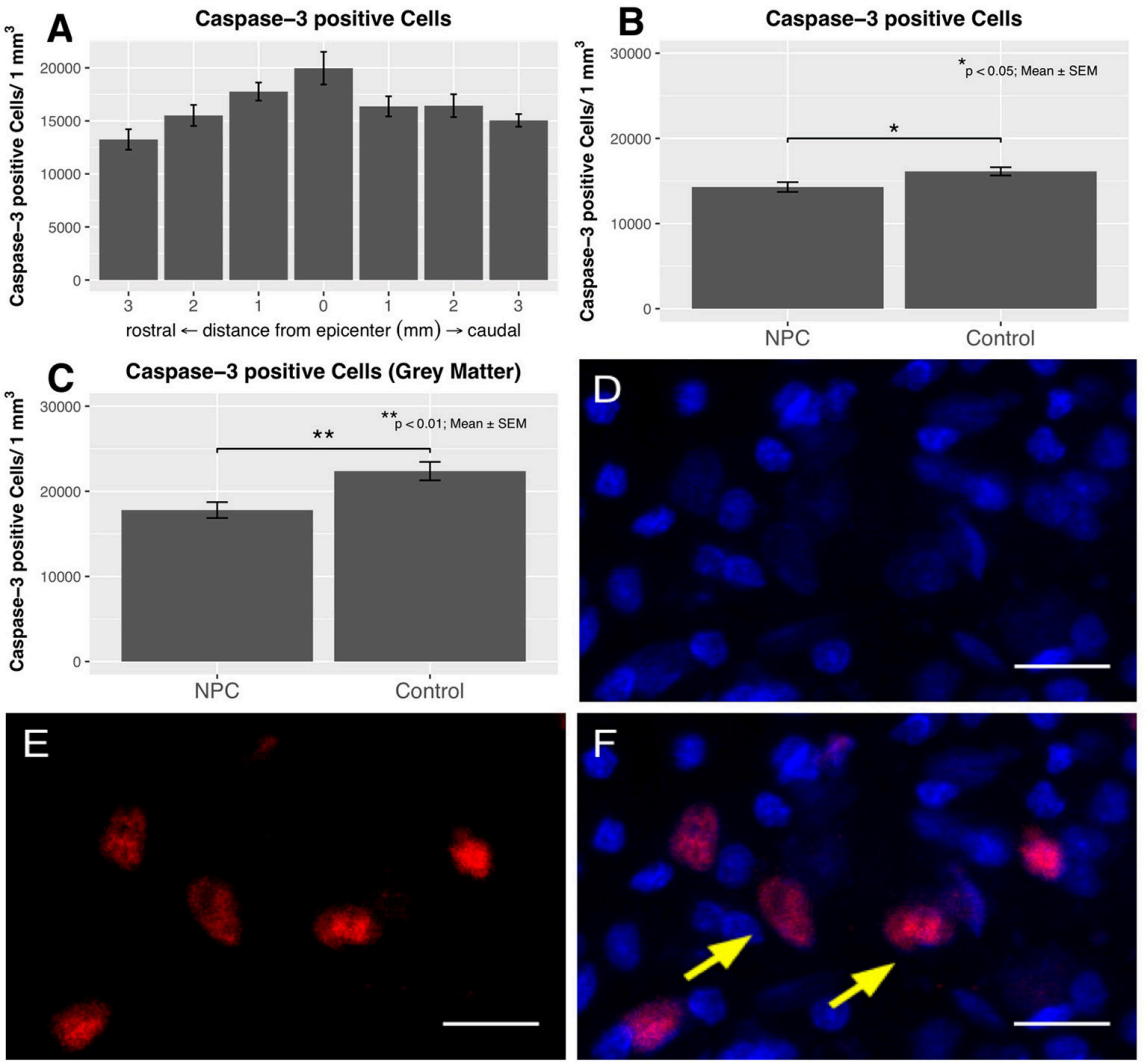
The distribution of caspase-3-positive cells shows an accumulation of apoptotic cells at the epicenter in injured control animals with apoptotic cell numbers steadily decreasing toward the periphery (*n* = 6). **(B)** The quantification of caspase-3-positive cell numbers yields a significant reduction in the NPC group compared to the control group (*n* = 6–7). **(C)** Apoptotic cell counts were generally higher in the gray matter and were also significantly reduced in the NPC group compared to the control group. **(D–F)** Caspase-3-positive cells (red) with additional DAPI-expression (blue) were counted as apoptotic cells, as indicated by the yellow arrows (scale bars = 15 μm).

Apoptotic cells were quantified by caspase-3 staining (control, *n* = 6; NPC, *n* = 7), both in the entire spinal cord and the gray matter (Figures 7D–F). The distribution of apoptotic cells in the chronic phase following SCI shows a pattern similar to that of iNOS-positive cells with a quantitative peak at the epicenter and a gradual decline toward the periphery (Figure 7A). In injured animals of the control group, 19,968 ± 1,538 caspase-3-positive cells were counted per mm^3^ at the epicenter. At 3 mm rostral and caudal of the lesion, apoptotic cell counts decreased to 13,245 ± 964 and 15,050 ± 596, respectively. Averaged per group, 16,130 ± 483 caspase-3-positive cells/mm^3^ were present in the control group compared to 14,291 ± 573 cells in the NPC group. This difference was statistically significant (*p* < 0.05; Figure 7B). Similar to the distribution of iNOS immunodensity, apoptotic cells were predominantly found in the gray matter. Quantification of the cells in the gray matter yielded a significant group difference with 17,792 ± 936 caspase-3-positive cells/mm^3^ in the NPC group vs. 22,366 ± 1,088 in the control group (*p* < 0.01; Figure 7C).

### Behavioral assessment

**Figure 8:**
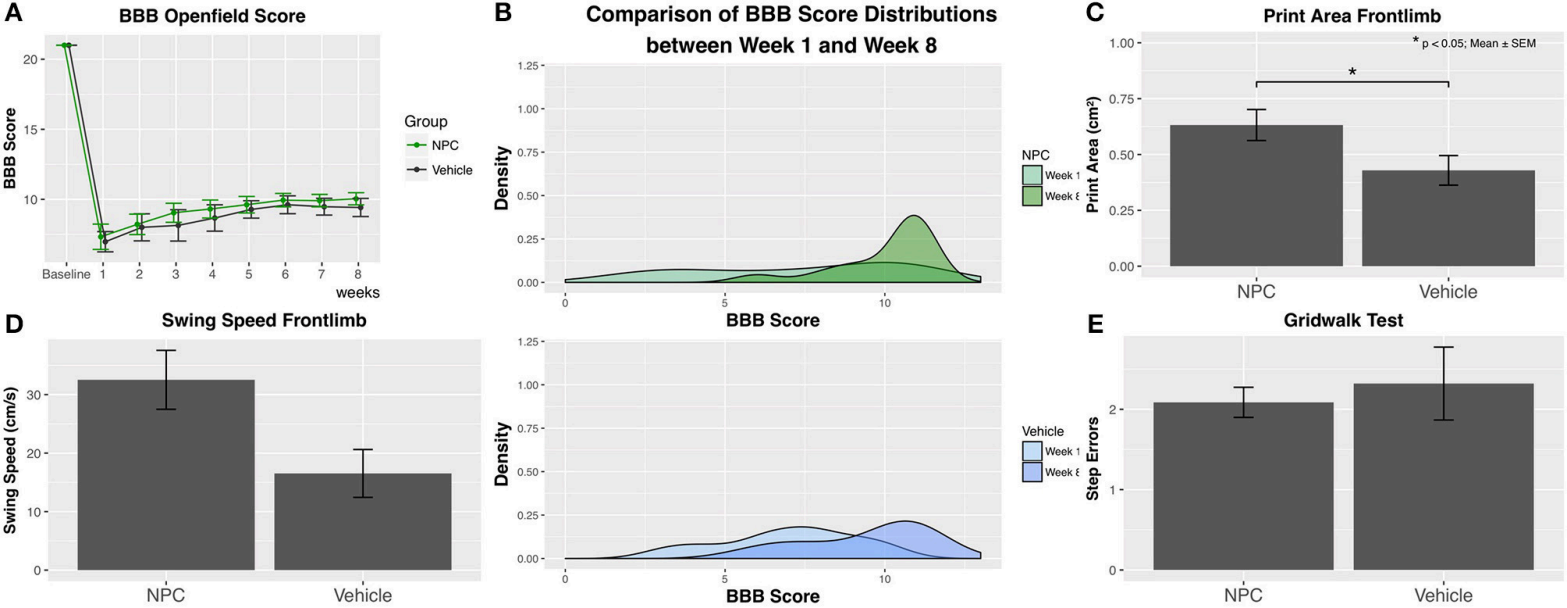
Functional recovery was weekly assessed using the BBB score, the CatWalk XT gait analysis, and the grid walk test (control, *n* = 9; NPC, *n* = 13). **(A)** Although group differences do not reach statistical significance, BBB scores are continuously higher in the NPC group compared to the control group at all observed time points. **(B)** The density curve, showing the distribution of BBB scores in each group for the first (week 1) and the last (week 8) time point, is clearly clinched toward higher scores in the NPC group. **(C)** The parameter “Print area” in the CatWalk XT analysis 8 weeks after SCI shows significantly better results (i.e., a larger forelimb area) in the NPC group compared to the control group**. (D)** The NPC group exhibits higher values in the forelimb parameter “Swing speed” of the CatWalk XT test without reaching statistical significance. **(E)** NPC-transplanted animals made only marginally fewer step errors in the grid walk test compared to control animals.

Functional recovery of hindlimbs was assessed using the BBB score. Baseline BBB values obtained before SCI showed with a score of 21 a normal neurologic function in all animals (control, *n* = 9; NPC, *n* = 13). One week after SCI, animals in both groups reached their lowest scores. During the following weeks, scores gradually improved to 10.04 ± 0.44 in the NPC group and to 9.41 ± 0.65 in the control group 8 weeks after SCI (Figure 8A). No animal in either of the groups reached a score higher than 12, reflecting the severe and long-lasting damage of cervical SCI, which massively impairs forelimb-hindlimb coordination. Although between-group differences for BBB scores did not reach statistical significance, the scores in the NPC group were continuously higher compared to those in the control group. The density distribution of BBB scores showed 8 weeks after SCI a clearly clinched curve for the NPC group compared to the control group, indicating that it was more likely for transplanted animals to reach a score above 9 (i.e., weight-bearing) (Figure 8B). NPC transplantation may foster a faster recovery since mean BBB scores above 9 were reached after 3 weeks in the NPC group compared to 5 weeks in the control group.

Locomotor regeneration of forelimbs was evaluated by the CatWalk XT analysis of footprints and kinematic motion in all animals (control, *n* = 9; NPC, *n* = 13). Considerable forelimb spasticity was observed in all injured animals. However, the CatWalk XT analysis of the forelimb shows 8 weeks after SCI a significantly larger footprint area, i.e., better results in the group receiving NPCs compared to the control group (NPC, 0.632 cm^2^; control, 0.429 cm^2^; *p* = 0.042; Figure 8C). Evaluation of the dynamic gait parameter “swing speed” that measures the velocity of a paw between ground contacts demonstrates higher values in the NPC group (32.53 cm/s) compared to the control group (22.22 cm/s), although this trend did not reach statistical significance (Figure 8D).

Fine motor control seemed to be not affected by stem cell transplantation with NPC-transplanted animals committing only marginally fewer step errors in the grid walk test compared to control animals (NPC, 2.087 ± 0.187; control, 2.320 ± 0.454; Figure 8E).

## Discussion

This study was designed to examine effects of neural stem cell transplantation on the immune environment in chronic stages of cervical spinal cord injury. The inflammatory response likely plays a pivotal role in the pathophysiology of SCI and the neuroregeneration after SCI (5, 8). Post-traumatic inflammation is not self-limiting and still detectable after months and years (6, 7, 40). Accordingly, as an evidence for this chronic inflammation, we were able to verify the presence of M1 macrophages, T lymphocytes, and apoptotic cells in substantial numbers in the perilesional area 8 weeks after SCI. In this chronic stage, astrogliosis and a cystic cavity were also present in injured animals. The transplanted NPCs showed long-term survival and substantial differentiation along the oligodendroglial lineage, as reported by others (31, 41).

Interestingly, transplantation of NPCs led to significantly lower numbers of M1 macrophages as well as a significantly lower iNOS immunodensity of the whole spinal cord and its gray matter. Moreover, significantly less apoptotic cells could be observed in NPC-transplanted animals compared to the control group. In addition, we could detect a non-significant trend showing smaller cystic cavities and better functional recoveries (i.e., higher BBB scores) in NPC-treated animals.

Macrophages are a decisive immune cell population in chronic inflammatory stages of SCI (12). Their functional state, often described as a pro-inflammatory M1 phenotype or an anti-inflammatory and pro-reparative M2 phenotype, has a great impact on regenerating neurons and their surrounding tissue (12, 16). Kigerl et al. (12) suggest that lesion-derived factors might influence the differentiation of macrophages, down-regulating M2 macrophages while promoting M1 phenotypes. Pharmacological therapies aiming at promoting a shift from M1 to M2 activation are reported to ameliorate the overall inflammatory response and lead to improved locomotor function (42, 43). As previously reported in other models, we uniformly observed a long-lasting presence of macrophages in our contusion-compression model of cervical SCI in rats (7, 16). M1 macrophages accumulated in the epicenter of the lesion with a steady decline in numbers toward the periphery. It has been shown that transplantation of stem cells can lead to a reduction of pro-inflammatory M1 macrophages in acute and sub-acute post-traumatic stages (20, 27, 44). In our study, we could show that cell numbers of M1 macrophages were significantly reduced in NPC-transplanted rats compared to the control group even in the chronic phase, 8 weeks after SCI. Possible mechanisms involve the down-regulation of pro-inflammatory cytokines, secretion of trophic factors, and modulation of the perilesional milieu. While M1 macrophages are assumed to persist after SCI, the number of M2 is reported to decline to normal levels before entering the chronic phase (12). We observed only very low numbers of M2 macrophages 8 weeks after SCI. Thus, the transplantation of NPCs did not have verifiable effects on the presence of M2 macrophages in the chronic stage. Collectively, our results suggest that transplantation of NPCs can attenuate M1 activation but cannot sustain or induce M2 activation in chronic stages.

Reactive astrogliosis is a characteristic response to injuries of the central nervous system (45). GFAP-expressing reactive astrocytes, while arguably being beneficial in the acute stage by walling off the lesion and limiting secondary degeneration, become an obstacle for regenerating neurons in the chronic phase due to the secretion of inhibitory molecules and astroglial scarring (46–49). In our study, we found a significant reduction of GFAP-positive astrogliosis in the NPC-transplanted group compared to the control group. A similar modulating effect of NPC transplantation on reactive astrogliosis in chronic stages of a cervical contusion/compression SCI model was shown by Wilcox et al. (25). These findings are of particular interest, as glial scarring is closely associated with inhibition of both axonal growth and neural plasticity (48). Modulating astrogliosis and inflammation in chronic stages could be of considerable therapeutic value, as studies indicate that even chronically injured neurons might be able to regenerate axons (50, 51).

Apoptotic cell death is a common feature of traumatic insults to the CNS. Many studies demonstrate post-traumatic apoptosis following SCI and decode signaling pathways (52–55). While necrosis seems to be present mainly in the early phase of SCI, apoptosis can be observed for weeks and at great distances from the epicenter of the traumatic lesion (56). We could confirm the presence of high numbers of apoptotic cell bodies 8 weeks after SCI. Their prevalence was highest at the epicenter with a continuous decrease toward the periphery, and apoptotic cells were still detectable 3 mm rostral and caudal of the lesion. The quantity of caspase-3-positive cell bodies suggesting apoptotic activity was significantly lower in the NPC group compared to the control group. Apoptosis is an important functional parameter, as apoptotic cells are closely related to neuronal and axonal damage and decay, tissue degeneration, and neurological dysfunction (52, 56). Transplantation of NPCs may have mitigated the post-traumatic hostile milieu, thus leading to a reduced number of cells undergoing apoptosis. Therefore, modulation of apoptotic activity might be a target to influence neuroregeneration even in chronic stages of SCI.

A limitation of our study is the substantial amount of GFP-positive NPCs that survived after transplantation but did not differentiate along either the oligodendroglial or neuronal lineage according to our quantification of APC+/NeuN+ stainings. As mentioned above, NPCs were characterized prior to transplantation by colocalization with the NPC-marker Nestin (GFP+/nestin+/DAPI+), however, an orthogonal measurement of such markers 6 weeks after transplantation was not performed. We rather proceeded based on the findings of other experiments that a vast part of precursor cells retains predominately undifferentiated features, as shown for example in a study by Cusimano et al. (44). It is however well documented that even undifferentiated precursor cells, solely expressing GFP, can greatly influence the injury environment and the secondary response after CNS trauma such as spinal cord injury and stroke (57).

In conclusion, our data indicate that transplantation of NPCs may attenuate the immune environment after cervical SCI even in chronic injury stages. Furthermore, the amount of pro-inflammatory M1 macrophages, as well as the immunodensity of their marker enzyme iNOS, are significantly reduced in the NPC-transplanted group 8 weeks after SCI, which is a finding of particular importance. An altered microenvironment in animals with NPC transplantation might have induced the significant reduction of reactive astrogliosis and post-traumatic apoptosis in our study. Finally, we could observe slight improvement of functional recovery in NPC-transplanted animals as well as a trend toward a reduction in cavity size.

## Ethics statement

The study was approved by the Animal Care Committee of Heidelberg University, and the government of Baden-Württemberg, Germany (G-211/15).

## Author contributions

LR and AY both equally share the first authorship, wrote the application, performed cell culture- and animal experiments, assessed and analyzed histology data, wrote the manuscript. MS performed animal experiments, neurobehavioral tests, corrected the manuscript. GZ performed animal experiments, neurobehavioral tests, assessed and analyzed histology data. TS provided and cultivated the stem cells, corrected the manuscript. AU supervised the project, corrected the manuscript. KZ (project leader) created and funded the project idea, established methods, trained co-workers in the methods (trauma, behavioral tests), corrected the manuscript.

### Conflict of interest statement

The authors declare that the research was conducted in the absence of any commercial or financial relationships that could be construed as a potential conflict of interest.

## Abbreviations

APC: Adenomatous Polyposis Coli
BBB: Basso, Beattie and Bresnahan
bFGF: Basic Fibroblast Growth Factor
CD206: Cluster of Differentiation 206
CD3: Cluster of Differentiation 3
CNS: Central Nervous System
DAPI: 4',6-diamidino-2-phenylindole
EGF: Epidermal Growth Factor
GFAP: Glial Fibrillary Acidic Protein
GFP: Green Fluorescent Protein
Iba1: Ionized Calcium-Binding Adapter Molecule 1
iNOS: Inducible Nitric Oxide Synthase
NPCs: Neural Precursor Cells
PBS: Phosphate-Buffered Saline
PDGF-AA: Platelet-Derived Growth Factor AA
SCI: Spinal Cord Injury.

## References

[1] National Spinal Cord Injury Statistical Center Annual Statistical Report (2016).

[2] Ruff CA Wilcox JT Fehlings MG. Cell-based transplantation strategies to promote plasticity following spinal cord injury. Exp Neurol. (2012) 235:78–90. 10.1016/j.expneurol.2011.02.010 21333647

[3] Bowes AL Yip PK. Modulating inflammatory cell responses to spinal cord injury: all in good time. J Neurotrauma (2014) 31:1753–66. 10.1089/neu.2014.3429 24934600

[4] Hausmann ON. Post-traumatic inflammation following spinal cord injury. Spinal cord (2003) 41:369–78. 10.1038/sj.sc.3101483 12815368

[5] Donnelly DJ Popovich PG. Inflammation and its role in neuroprotection, axonal regeneration and functional recovery after spinal cord injury. Exp Neurol. (2008) 209:378–88. 10.1016/j.expneurol.2007.06.009 17662717 PMC2692462

[6] Fleming JC Norenberg MD Ramsay DA Dekaban GA Marcillo AE Saenz AD. The cellular inflammatory response in human spinal cords after injury. Brain (2006) 129(Pt 12):3249–69. 10.1093/brain/awl296 17071951

[7] Pruss H Kopp MA Brommer B Gatzemeier N Laginha I Dirnagl U. Non-resolving aspects of acute inflammation after spinal cord injury (SCI): indices and resolution plateau. Brain Pathol. (2011) 21:652–60. 10.1111/j.1750-3639.2011.00488.x 21418368 PMC8094060

[8] Schwab JM Zhang Y Kopp MA Brommer B Popovich PG. The paradox of chronic neuroinflammation, systemic immune suppression, autoimmunity after traumatic chronic spinal cord injury. Exp Neurol. (2014) 258:121–9. 10.1016/j.expneurol.2014.04.023 25017893 PMC4099970

[9] Jones TB. Lymphocytes and autoimmunity after spinal cord injury. Exp Neurol. (2014) 258:78–90. 10.1016/j.expneurol.2014.03.003 25017889

[10] Sroga JM Jones TB Kigerl KA McGaughy VM Popovich PG. Rats and mice exhibit distinct inflammatory reactions after spinal cord injury. J Comp Neurol. (2003) 462:223–40. 10.1002/cne.10736 12794745

[11] Kigerl KA McGaughy VM Popovich PG. Comparative analysis of lesion development and intraspinal inflammation in four strains of mice following spinal contusion injury. J Comp Neurol. (2006) 494:578–94. 10.1002/cne.20827 16374800 PMC2655318

[12] Kigerl KA Gensel JC Ankeny DP Alexander JK Donnelly DJ Popovich PG. Identification of two distinct macrophage subsets with divergent effects causing either neurotoxicity or regeneration in the injured mouse spinal cord. J Neurosci. (2009) 29:13435–44. 10.1523/JNEUROSCI.3257-09.2009 19864556 PMC2788152

[13] Popovich PG Guan Z Wei P Huitinga I van Rooijen N Stokes BT. Depletion of hematogenous macrophages promotes partial hindlimb recovery and neuroanatomical repair after experimental spinal cord injury. Exp Neurol. (1999) 158:351–65. 10415142 10.1006/exnr.1999.7118

[14] Popovich PG Guan Z McGaughy V Fisher L Hickey WF Basso DM. The neuropathological and behavioral consequences of intraspinal microglial/macrophage activation. J Neuropathol Exp Neurol. (2002) 61:623–33. 10.1093/jnen/61.7.623 12125741

[15] Gordon S. Alternative activation of macrophages. Nat Rev Immunol. (2003) 3:23–35. 10.1038/nri978 12511873

[16] David S Kroner A. Repertoire of microglial and macrophage responses after spinal cord injury. Nat Rev Neurosci (2011) 12(7):388–99. 10.1038/nrn3053 21673720

[17] Gensel JC Zhang B. Macrophage activation and its role in repair and pathology after spinal cord injury. Brain Res. (2015) 1619:1–11. 10.1016/j.brainres.2014.12.045 25578260

[18] Ahuja CS Fehlings M. Concise review: Bridging the gap: Novel neuroregenerative and neuroprotective strategies in spinal cord injury. Stem Cells Transl Med. (2016) 5:914–24. 10.5966/sctm.2015-0381 27130222 PMC4922857

[19] Madhavan L Ourednik V Ourednik J. Neural stem/progenitor cells initiate the formation of cellular networks that provide neuroprotection by growth factor-modulated antioxidant expression. Stem Cells (2008) 26:254–65. 10.1634/stemcells.2007-0221 17962704

[20] Nakajima H Uchida K Guerrero AR Watanabe S Sugita D Takeura N. Transplantation of mesenchymal stem cells promotes an alternative pathway of macrophage activation and functional recovery after spinal cord injury. J Neurotrauma (2012) 29:1614–25. 10.1089/neu.2011.2109 22233298 PMC3353766

[21] Lepore AC Bakshi A Swanger SA Rao MS Fischer I. Neural precursor cells can be delivered into the injured cervical spinal cord by intrathecal injection at the lumbar cord. Brain Res. (2005) 1045:206–16. 10.1016/j.brainres.2005.03.050 15910779

[22] Mothe AJ Tator CH. Review of transplantation of neural stem/progenitor cells for spinal cord injury. Int J Dev Neurosci. (2013) 31:701–13. 10.1016/j.ijdevneu.2013.07.004 23928260

[23] Mothe AJ Tator CH. Advances in stem cell therapy for spinal cord injury. J Clin Invest (2012) 122:3824–34. 10.1172/JCI64124 23114605 PMC3484454

[24] Hawryluk GW Spano S Chew D Wang S Erwin M Chamankhah M. An examination of the mechanisms by which neural precursors augment recovery following spinal cord injury: a key role for remyelination. Cell Transplant. (2014) 23:365–80. 10.3727/096368912X662408 23363615

[25] Wilcox JT Satkunendrarajah K Zuccato JA Nassiri F Fehlings MG. Neural precursor cell transplantation enhances functional recovery and reduces astrogliosis in bilateral compressive/contusive cervical spinal cord injury. Stem Cells Transl Med. (2014) 3:1148–59. 10.5966/sctm.2014-0029 25107585 PMC4181397

[26] Hawryluk GW Mothe A Wang J Wang S Tator C Fehlings MG An *in vivo* characterization of trophic factor production following neural precursor cell or bone marrow stromal cell transplantation for spinal cord injury. Stem Cells Dev. (2012) 21:2222–38. 10.1089/scd.2011.0596 22085254 PMC3411361

[27] Cheng Z Zhu W Cao K Wu F Li J Wang G. Anti-inflammatory mechanism of neural stem cell transplantation in spinal cord injury. Int J Mol Sci. (2016) 17:E1380. 10.3390/ijms17091380 27563878 PMC5037660

[28] Zweckberger K Liu Y Wang J Forgione N Fehlings MG Synergetic use of neural precursor cells and self-assembling peptides in experimental cervical spinal cord injury. J Vis Exp. (2015) 96:e52105 10.3791/52105 PMC4354669 25742521

[29] Iwasaki M Wilcox JT Nishimura Y Zweckberger K Suzuki H Wang J. Synergistic effects of self-assembling peptide and neural stem/progenitor cells to promote tissue repair and forelimb functional recovery in cervical spinal cord injury. Biomaterials (2014) 35:2617–29. 10.1016/j.biomaterials.2013.12.019 24406216

[30] Zweckberger K Ahuja CS Liu Y Wang J Fehlings MG. Self-assembling peptides optimize the post-traumatic milieu and synergistically enhance the effects of neural stem cell therapy after cervical spinal cord injury. Acta Biomater. (2016) 42:77–89. 10.1016/j.actbio.2016.06.016 27296842

[31] Karimi-Abdolrezaee S Eftekharpour E Wang J Morshead CM Fehlings MG. Delayed transplantation of adult neural precursor cells promotes remyelination and functional neurological recovery after spinal cord injury J Neurosci. (2006) 26:3377–89. 10.1523/JNEUROSCI.4184-05.2006 16571744 PMC6673854

[32] Karimi-Abdolrezaee S Eftekharpour E Wang J Schut D Fehlings MG. Synergistic effects of transplanted adult neural stem/progenitor cells, chondroitinase, and growth factors promote functional repair and plasticity of the chronically injured spinal cord. J Neurosci. (2010) 30:1657–76. 10.1523/JNEUROSCI.3111-09.2010 20130176 PMC6634011

[33] Liu Y Ye H Satkunendrarajah K Yao GS Bayon Y Fehlings MG. A self-assembling peptide reduces glial scarring, attenuates post-traumatic inflammation and promotes neurological recovery following spinal cord injury. Acta Biomater. (2013) 9:8075–88. 10.1016/j.actbio.2013.06.001 23770224

[34] Basso DM Beattie MS Bresnahan JC. A sensitive and reliable locomotor rating scale for open field testing in rats. J Neurotrauma (1995) 12:1–21. 10.1089/neu.1995.12.1 7783230

[35] Hamers FP Koopmans GC Joosten EA. CatWalk-assisted gait analysis in the assessment of spinal cord injury. J Neurotrauma (2006) 23:537–48. 10.1089/neu.2006.23.537 16629635

[36] Metz GA Merkler D Dietz V Schwab ME Fouad K. Efficient testing of motor function in spinal cord injured rats. Brain Res. (2000) 883:165–77. 10.1016/S0006-8993(00)02778-5 11074045

[37] R Core Team R: A Language and Environment for Statistical Computing.: R Foundation for Statistical Computing, Vienna, Austria (2016).

[38] Sofroniew MV Vinters HV. Astrocytes: biology and pathology. Acta Neuropathol. (2010) 119:7–35. 10.1007/s00401-009-0619-8 20012068 PMC2799634

[39] Kjell J Olson L. Rat models of spinal cord injury: from pathology to potential therapies. Dis Model Mech. (2016) 9:1125–37. 10.1242/dmm.025833 27736748 PMC5087825

[40] Beck KD Nguyen HX Galvan MD Salazar DL Woodruff TM Anderson AJ. Quantitative analysis of cellular inflammation after traumatic spinal cord injury: evidence for a multiphasic inflammatory response in the acute to chronic environment. Brain (2010) 133:433–47. 10.1093/brain/awp322 20085927 PMC2858013

[41] Parr AM Kulbatski I Zahir T Wang X Yue C Keating A. Transplanted adult spinal cord-derived neural stem/progenitor cells promote early functional recovery after rat spinal cord injury. Neuroscience (2008) 155:760–70. 10.1016/j.neuroscience.2008.05.042 18588947

[42] Ji XC Dang YY Gao HY Wang ZT Gao M Yang Y. Local injection of lenti-BDNF at the lesion site promotes M2 macrophage polarization and inhibits inflammatory response after spinal cord injury in mice. Cell Mol Neurobiol. (2015) 35:881–90. 10.1007/s10571-015-0182-x 25840805 PMC11486196

[43] Zhang Q Bian G Chen P Liu L Yu C Liu F. Aldose reductase regulates microglia/macrophages polarization through the cAMP response element-binding protein after spinal cord injury in mice. Mol Neurobiol. (2016) 53:662–76. 10.1007/s12035-014-9035-8 25520004

[44] Cusimano M Biziato D Brambilla E Donegà M Alfaro-Cervello C Snider S. Transplanted neural stem/precursor cells instruct phagocytes and reduce secondary tissue damage in the injured spinal cord. Brain (2012) 135:447–60. 10.1093/brain/awr339 22271661 PMC3558737

[45] Karimi-Abdolrezaee S Billakanti R. Reactive astrogliosis after spinal cord injury-beneficial and detrimental effects. Mol Neurobiol. (2012) 46:251–64. 10.1007/s12035-012-8287-4 22684804

[46] Bush TG Puvanachandra N Horner CH Polito A Ostenfeld T Svendsen CN. Leukocyte infiltration, neuronal degeneration, and neurite outgrowth after ablation of scar-forming, reactive astrocytes in adult transgenic mice. Neuron (1999) 23:297–308. 10399936 10.1016/s0896-6273(00)80781-3

[47] Faulkner JR Herrmann JE Woo MJ Tansey KE Doan NB Sofroniew MV. Reactive astrocytes protect tissue and preserve function after spinal cord injury. J Neurosci. (2004) 24:2143–55. 10.1523/JNEUROSCI.3547-03.2004 14999065 PMC6730429

[48] Silver J Miller JH. Regeneration beyond the glial scar. Nat Rev Neurosci. (2004) 5:14–156. 10.1038/nrn1326 14735117

[49] Davies SJ Fitch MT Memberg SP Hall AK Raisman G Silver J. Regeneration of adult axons in white matter tracts of the central nervous system. Nature (1997) 390:680–3. 10.1038/37776 9414159

[50] Houle JD. Demonstration of the potential for chronically injured neurons to regenerate axons into intraspinal peripheral nerve grafts. Exp Neurol. (1991) 113:1–9. 2044676 10.1016/0014-4886(91)90139-4

[51] Kwon BK Liu J Messerer C Kobayashi NR McGraw J Oschipok L. Survival and regeneration of rubrospinal neurons 1 year after spinal cord injury. Proc Natl Acad Sci U S A. (2002) 99:3246–51. 10.1073/pnas.052308899 11867727 PMC122504

[52] Liu XZ Xu XM Hu R Du C Zhang SX McDonald JW. Neuronal and glial apoptosis after traumatic spinal cord injury. J Neurosci. (1997) 17:5395–406. 9204923 10.1523/JNEUROSCI.17-14-05395.1997 PMC6793816

[53] Yong C Arnold PM Zoubine MN Citron BA Watanabe I Berman NE. Apoptosis in cellular compartments of rat spinal cord after severe contusion injury. J Neurotrauma (1998) 15:459–72. 10.1089/neu.1998.15.459 9674550

[54] Li GL Brodin G Farooque M Funa K Holtz A Wang WL. Apoptosis and expression of Bcl-2 after compression trauma to rat spinal cord. J Neuropathol Exp Neurol. (1996) 55:280–9. 8786386 10.1097/00005072-199603000-00003

[55] Casha S Yu WR Fehlings MG. Oligodendroglial apoptosis occurs along degenerating axons and is associated with FAS and p75 expression following spinal cord injury in the rat. Neuroscience (2001) 103:203–18. 10.1016/S0306-4522(00)00538-8 11311801

[56] Beattie MS Farooqui AA Bresnahan JC. Review of current evidence for apoptosis after spinal cord injury. J Neurotrauma (2000) 17:915–25. 10.1089/neu.2000.17.915 11063057

[57] Giusto E Donega M Cossetti C Pluchino S. Neuro-immune interactions of neural stem cell transplants: from animal disease models to human trials. Exp Neurol. (2014) 260:19–32. 10.1016/j.expneurol.2013.03.009 23507035 PMC4162671

